# Neonatal presentation of Loeys-Dietz syndrome: two case reports and review of the literature

**DOI:** 10.1186/s13052-022-01281-y

**Published:** 2022-06-06

**Authors:** Francesco Baldo, Laura Morra, Agnese Feresin, Flavio Faletra, Yasmin Al Naber, Luigi Memo, Laura Travan

**Affiliations:** 1 University of Trieste, Department of Medicine, Surgery and Health Sciences, Trieste, Italy; 2 Department of Genetics, Institute for Maternal and Child Health IRCCS “Burlo Garofolo”, Trieste, Italy; 3 Clinical Genetics Outpatient Service, Pediatric Unit, “San Giovanni e Paolo” Hospital, Venice, Italy; 4 Department of Neonatology, Institute for Maternal and Child Health IRCCS “Burlo Garofolo”, Trieste, Italy

**Keywords:** Loeys-Dietz syndrome, Newborn, Aneurysm, Connective tissue disorder

## Abstract

**Background:**

Loeys-Dietz syndrome (LDS) is a rare connective tissue disorder characterized by cardiovascular manifestations, especially aortic dilatations and arterial tortuosity, craniofacial and skeletal features, joint laxity or contractures, skin abnormalities, hypotonia and motor delay. Its diagnosis is established by the identification of a pathogenic variant in *TGFBR1, TGFBR2, SMAD2, SMAD3, TGFB2* or *TGFB3* genes. In newborns and toddlers, vascular complications such as aneurism rupture, aortic dissection, and intracerebral incidents, can occur already in the weeks of life. To avoid these events, it is crucial to precociously identify this condition and to start an apunderwent a surgical procedurepropriate treatment which, depending on the severity of the vascular involvement, might be medical or surgical.

**Case presentation:**

We report two cases of Loeys-Dietz syndrome precociously diagnosed. The first describes a male, born at 38 + 1 weeks of gestation, with hypotonia, joint hypermobility, arachnodactyly, and fingers joint contractures, as well as senile appearance and facial dysmorphisms. In the suspect of a connective tissue disorder, an echocardiography was performed and revealed an aortic root dilatation of 13 mm (Z score + 3). A trio based Whole Exome Sequencing found a novel de novo variant in the *TGFBR2* gene. Despite the onset of a low-dose angiotensin receptor blocker therapy, the aneurysm progressed. The second case describes a female, born at 41 + 3 weeks of gestation. During the neonatal examination a cleft palate was noticed, as well as minor dysmorphisms. Since the family history was suspicious for connective tissue disorders, a genetic panel was performed and identified a pathogenetic variant in *TGFB3* gene. In this case, the echocardiography revealed no abnormalities.

**Conclusions:**

In addition to our cases, we identified 14 subjects with neonatal LDS in the medical literature. All of them had aortic involvement. Skeletal and face abnormalities, including eyes and palate malformations, were also highly frequent. Overall, 10 subjects required medical therapy to avoid aneurysm progression, and 8 patients underwent surgical procedures.

Benefits of an early diagnosis of LDS are various and imply a potential modification of the natural history of the disease with early interventions on its complications.

## Background

Loeys-Dietz syndrome (LDS) is an autosomal dominant connective tissue disorder described for the first time in 2005 [1]. This condition is typically characterized by a triad that consists of arterial tortuosity and aneurysms, hypertelorism and bifid uvula or cleft palate [2]. However, LDS’s phenotypic spectrum is highly variable, ranging from a precocious syndromic presentation in newborns and toddlers, with typical facial dysmorphisms and severe systemic features, to isolated aortic aneurysms incidentally discovered in adults.

Being the main causes of morbidity and early mortality in LDS, the cardiovascular manifestations are the key elements of this condition. Patients typically develop dilatations of the aorta and the pulmonary arteries, as well as a predisposition for aortic dissection. Arterial tortuosity is also present, and it is typically widespread, affecting a variety of aortic branches in the abdominal, pelvic, and intracranial area. It is estimated that around 50% of the subjects with LDS have an aneurysm distal from the aortic root that would have not been detected by echocardiography [3]. This extensive vascular involvement is a very distinct feature of LDS, and makes it differ from other connective tissue disorders, especially Marfan syndrome (MS). Furthermore, arterial tortuosity tends to appear early in LDS, so that it can be seen even on prenatal ultrasound. Indeed, an isolated aortic aneurysm detected in a fetus represents an indication to test for LDS [4]. Also, aortic dissections and rupture tend to develop with smaller aortic root’s dimensions compared to MS. Consequently, the mean age of death in LDS (26 years old) is significantly lower than MS [5]. As for the cardiac involvement, patent ductus arteriosus (PDA), atrial septal defects, mitral valve prolapse, and bicuspid aortic valve are also relatively common finding.

Craniofacial abnormalities include craniosynostosis, micro-retrognathia, cleft palate and bifid uvula, while eyes’ involvement is usually characterized by hypertelorism, strabismus and blue sclerae. Retinal hemorrhages and retinal detachment are also associated with this condition, while lens dislocation is not as typical as in MS. Generally, facial features in LDS patients are usually associated with a more aggressive and severe aortic disease [6].

The skeletal features of LDS are those typically associated with connective tissue disorders and include joint laxity (as well as congenital hip dislocation and recurrent joint subluxations) and joint contractures (camptodactyly, clenched hands, clubfeet), arachnodactyly and pectus excavatum or carinatum. Spinal abnormalities include cervical spine malformations, especially C2-C3 subluxations, spinal stenosis, and early-onset scoliosis [7].

Skin findings are similar to those in Ehlers-Danlos syndrome, including velvety and translucent skin, easy bruising and dystrophic scarring. Hernias occur at higher rate than the general population. Neurodevelopmental abnormalities are frequent, especially hypotonia and motor delays. Feeding issues are also common [8]. Lastly, patients with LDS are more prone to develop allergic diseases (such asthma, food allergies) and inflammatory bowel disease than the general population.

LDS is suspected by the recognition of both an aortic root enlargement or type A dissection and compatible systemic futures. The molecular diagnosis is established by the identification of a pathogenic variant in *TGFBR1, TGFBR2, SMAD2, SMAD3, TGFB2* or *TGFB3* genes, encoding for components cooperating within the TGF-beta receptor signaling cascade to regulate cell differentiation and the development of many tissues, including bone, cartilage, blood vessels, heart [9]. Heterozygous, loss-of function variants in *TGFBR1* and *TGFBR2* are the most frequently identified in LDS patients and are responsible for up to 80% of the cases. Until now, there is no evidence of specific genotype-phenotype correlations.

LDS differential diagnosis includes other hereditary connective tissue disorders, mainly Marfan syndrome, but also Beals syndrome, Ehlers-Danlos syndrome, and Shprintzen-Goldberg syndrome.

Compared to Marfan syndrome, LDS patients usually don’t show significant long bone overgrowth and lens dislocation. On the other hand, Beals syndrome is typically not characterized by vascular abnormalities [10].

Currently there are not standardized treatments and consensus protocols for LDS patients, and only few clinical trials have been performed. Most of the information available can be inferred from studies regarding other connective tissue disorders, especially MS. Various medical approaches have been reported in the attempt to slow down the development of vascular abnormalities in these genetic conditions, including beta-blockers, ACE-inhibitors, and angiotensin receptor blockers (ARBs), especially losartan. Among patients with MS, beta-blockers and losartan showed efficacy in reducing aortic root dilatations and need for surgical interventions, especially starting therapies at a young age [11, 12]. When administered as a single therapy, no significant difference has been found comparing the two drug classes; however, the combination of a beta-blocker and losartan have showed to be more efficient than the beta-blocker alone [13, 14]. These clinical data confirm the positive expectations towards losartan, which previously showed the ability to fully correct vascular abnormalities on mouse models with MS [15]. In light of these findings, although beta-blockers remain the first line therapy for MS, some authors currently advocate a precocious losartan treatment as a valuable alternative [16].

Finally, dexamethasone has shown some results on in vitro studies, improving the extracellular matrix deposition in fibroblasts with TGFBR1 and TGFBR2 mutations [17, 18]. However, clinical studies to confirm the possible benefits in vivo have not been conducted yet.

In case of severe, rapidly progressing aneurysms, that do not respond to the medical therapy or that put the patient’s life at risk, an early and aggressive surgical intervention is required. Valve spearing procedures are generally preferred since they preclude the need for chronic anticoagulation.

In newborns, toddlers, and young children in general, especially with severe craniofacial features, aneurism’s rupture, aortic dissection, and intracerebral incidents can occur already in first months of life. Therefore, specific thresholds, based on age and clinical symptoms, have been established to plan surgical procedures (Table 1).

**Table 1:** Surgical recommendations in Loeys-Dietz syndrome (from Williams et al., modified) [19]

Children		Adults
Severe craniofacial features	Mild craniofacial features	
1) Aortic root z-score > 3 or rapidly expanding (0.5 cm over 1 year)	1) Aortic root z-score > 4 or rapidly expanding (0.5 cm over 1 year)	1) Aortic root z-score > 4 or rapidly expanding (0.5 cm over 1 year)
2) Effort made to delay surgery until the annulus reaches 1.8 cm, allowing placement of a valve sparing graft to sufficient size to accommodate growth	2) Large size or rapid expansion of the descending aorta or other vessels	2) Descending thoracic aorta > 5 cm or rapidly expanding (0.5 cm over 1 year)
		3) Abdominal aorta > 4 cm or rapidly expanding (0.5 cm over 1 year)
		4) Rapid expansion of peripheral aneurysm

Apart from the vascular abnormalities, the remaining clinical features are managed according to the referring specialist.

### Case presentation

#### First clinical case

A male, naturally conceived, third-born child of a non-consanguineous couple with un unremarkable family history and healthy brothers and sisters, was born in a secondary-care hospital by vaginal delivery at 38 + 1 weeks of gestation after a dichorionic diamniotic twin pregnancy. Routine fetal ultrasound showed intrauterine growth restriction in both fetuses. Interestingly, the mother perceived a significant difference between the fetal movements of the two twins in the last weeks of pregnancy, with one being barely noticeable.

At birth, the patient Apgar score was 9 and 9 at respectively 1 and 5 minutes of life, due to persistence of hypotonia. The newborn’s weight was 2560 g (10° percentile), his length was 49 cm (25–50° percentile), and his head circumference was 35 cm (50–75° percentile). The female twin presented similar measurements, but she was not hypotonic, and her Apgar score was therefore 9 and 10.

The first neonatal visit confirmed the axial hypotonia, the joint hypermobility, the right congenital genu recurvatum (which had a spontaneous resolution within 12 hours), the arachnodactyly and the fingers joint contractures.

All the newborn’s screening tests (hearing, pulse oximetry, and inborn error of metabolism screening) were normal.

**Fig. 1:**
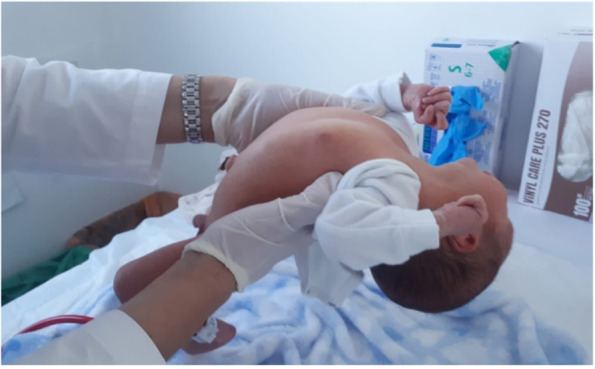
Patient 1: axial hypotonia

**Fig. 2:**
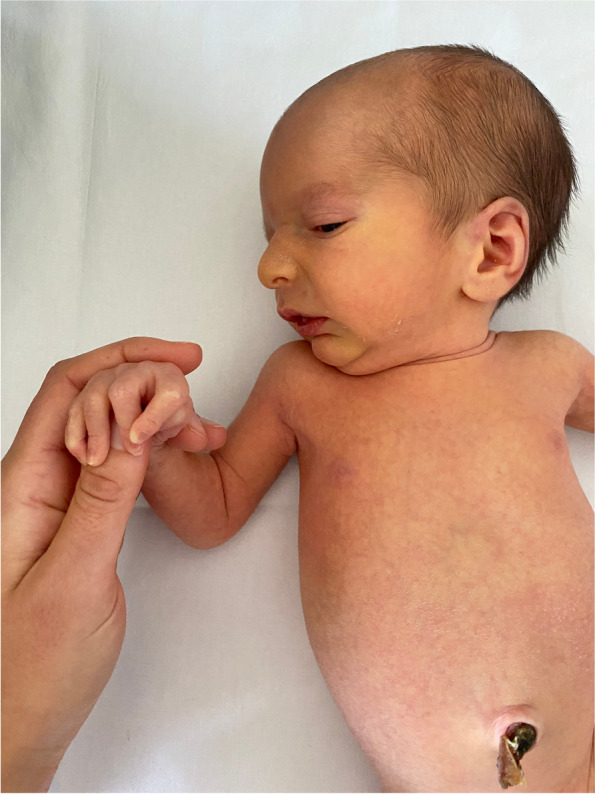
Patient 1: senile appearance

**Fig. 3:**
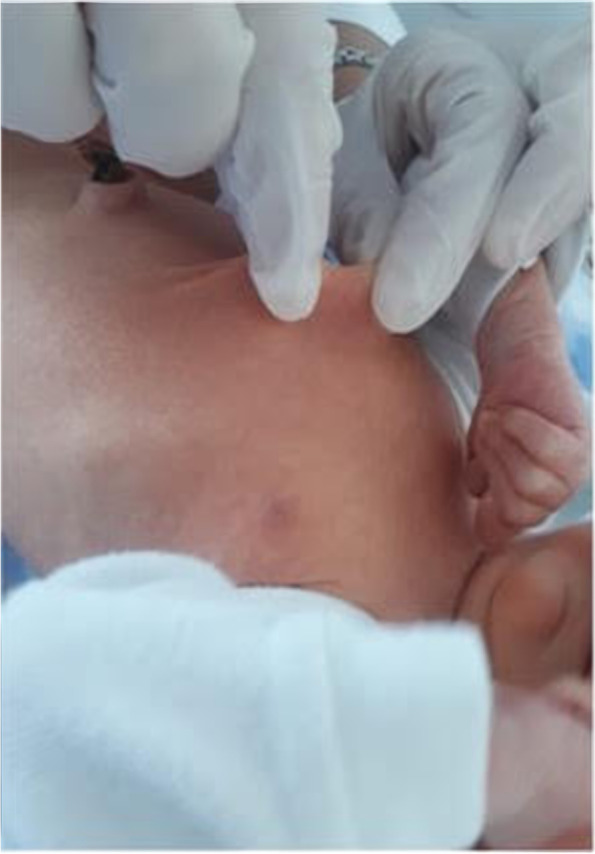
Patient 1: lax skin

The infant was referred to our tertiary care Department of Neonatology at 5 days of life for further evaluations. On physical examination, the newborn was in good general conditions. Axial hypotonia and poor head control were evident, as well as poor sucking (Fig. 1). The most feature was a peculiar senile appearance (Fig. 2). Several facial dysmorphisms were noticed, including hypertelorism, down-slanting palpebral fissures, ocular exotropia, arched nose with a bulbous nasal tip, high-vaulted palate, short philtrum, down-turned mouth with thin upper lip, micrognathia, and low set ears with evident lobes. Cleft palate was not present. Skin appeared lax and translucent (Fig. 3). Furthermore, the patient showed arachnodactyly, contracted finger joints, adduction deformity of the right thumb, clinodactyly and ulnar deviation of all hand fingers. Camptodactyly of the feet was also noticed with a tendency of toes flexion. Nails appeared dystrophic. No pterygia were noticed. The heart rhythm was regular without audible murmurs, and femoral pulses were palpable and symmetrical.

In consideration of this clinical phenotype, a congenital connective tissue disorder was suspected, and ophthalmic evaluation, echocardiography and abdominal ultrasound scan were requested. The ophthalmic evaluation confirmed the exotropia and did not find any other abnormalities, as well as the abdominal ultrasound. Instead, the echocardiography revealed an aortic root dilatation at the aortic sinus of 13 mm (Z score + 3) without aortic insufficiency. The remaining segments of the aorta appeared within limits. The patient also presented patent foramen ovale and ductus arteriosus, with a hemodynamically not significant left-to-right shunt. After the genetic evaluation, a trio based Whole Exome Sequencing (WES) was performed finding a novel, heterozygote, missense, de novo variant (c.1378C > G, p.(Arg460Gly) in the *TGFBR2* (NM_003242.5) gene.

Due to the substantial aortic root dilation and the precocity of the clinical finding, a strict cardiologic follow-up was arranged. At 5 months of life, the aortic dilation progressed to 18 mm (+ 5 mm from the first evaluation, Z score + 4.7 according to the Boston Children’s Hospital Z score or + 3.58 according to AJC 2010) [20, 21]. Therefore, as suggested by the current literature, low-dose angiotensin receptor blocker therapy (losartan) was started at 0.6 mg/kg/day, to prevent further dilatation and dissection of the aorta [22]. After an initial stabilization of the aortic dilation (20 mm at 7 months of age, + 3.2 Boston Children’s Hospital Z score), the aneurysm continued to enlarge and now, at 13 months of age, its diameter is 24.5 mm (+ 6.7 Boston Children’s Hospital Z score). In consideration of the echocardiographic progression, we will start a beta-blocker (nebivolol, 0.1 mg/Kg/die) and consider a surgical treatment.

Neurologically the patient showed steady improvements: his suction became efficient with benefit on his growth (50–75° percentile) and the hypotonia sensibly reduced, allowing the toddler to roll over and to sit with support and then crawl. Despite physiotherapy, his fingers motility remained limited, especially the full extension of the thumb and the third and fourth fingers.

A magnetic resonance angiography with three-dimensional reconstruction of the head, neck, chest, abdomen, and pelvis will be performed soon to identify the presence of other aneurysms throughout the aorta and arterial tree.

#### Second clinical case

A female newborn of non-consanguineous parents was born from vaginal delivery at 41 weeks + 3 days of gestation. Apgar score was 8, 10, 10 at respectively 1, 5 and 10 minutes of life. Her birth weight was 3470 g (50° percentile), her length was 53 cm (50° percentile) and hear head circumference measured 34 cm (10° percentile). During the neonatal examination a cleft palate, involving both the soft and hard tissue, was noticed, without any other abnormalities. The perinatal period was otherwise unremarkable, except for neonatal jaundice due to AB0 incompatibility that required phototherapy. All the newborn’s screening tests (hearing, pulse oximetry, and inborn error of metabolism screening) were normal.

The patient family history was relevant for the presence of an older brother with ligamentous hyperlaxity, overabundant skin, coetaneous xerosis, and slender fingers and toes, all which were identified at birth. At 2 months of age a neurological assessment was normal, with tone and strength adequate per age. Cardiological, audiological and ophthalmological evaluations were also performed and revealed no abnormalities. Ultimately, a genetic counseling advised against the execution of genetic tests, even if the infant’s mother presented ligamentous hyperlaxity too.

**Fig. 4:**
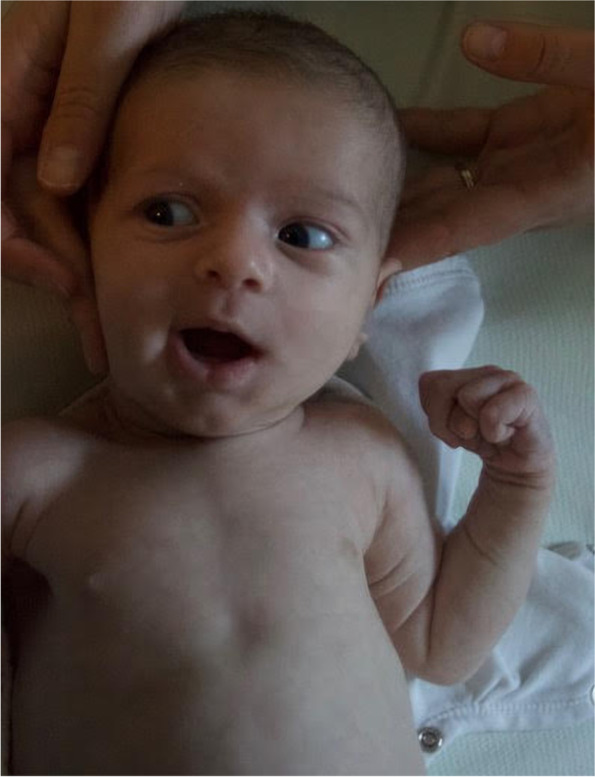
Patient 2: overall appearance

The baby girl was referred to our attention at 1 month of life. At that time, her weight was 4130 g (50–75 ° percentile), her length was 56.5 cm (about 90° percentile), and her head circumference was 38.7 cm (90° percentile). On physical examination minor dysmorphic were noticed (mild hypertelorism and micrognathia, everted ears and pectus excavatum), as well as the already known cleft palate (Fig. 4). The girl’s skin appeared dry and overabundant, without stretch marks, nor atrophic or hypertrophic scars. Slight joints’ laxity and arachnodactyly were also noticed.

An echocardiography was performed and resulted normal, without signs of aneurysms, valve insufficiency or structural abnormalities.

Contrary to the first genetic consult, in consideration of the suggestive patient’s family history, a connective disorder gene panel was performed. The test identified an already known, heterozygote, pathogenetic variant in *TGFB3* gene (c899G > A p.Arg300Gln) causative for Loeys-Dietz syndrome The segregation analysis revealed that the variant was inherited by the mother and also present in the patient’s brother confirming the diagnosis of Loeys-Dietz syndrome in them as well.

## Discussion

To identify all the cases of LDS recognized in the neonatal period, we performed a research in the medical literature through Pubmed and PMC using the keywords “Loeys-Dietz” plus “neonatal”, “newborn”, “toddler” and “infant”. 31 articles came out from Pubmed and 320 from PMC, but only 11 of them were covering the topic [23–31]. All of the compatible articles were case reports, except for a small case series. Two case reports discussed the clinical history of the same patient.

14 patients were identified from the literature, reaching, with the addition of the case reports described above, a total of 16 subjects (Table 2).

**Table 2:** Genetic variants, time of appearance of vascular abnormalities and original diagnostic suspect in the 16 patients identified with early diagnosis of Loeys Dietz syndrome

Report	Variant	Appearance of vascular abnormalities	Original diagnostic suspect
Choo JTL et al	1583G > A(R528H), exon 7, TGFBR2	2 months	Arthrogryposis
Ilyn VN et al	/	Prenatal ultrasound	/
Muramatsu Y et al	c.1370 T > A(M457L), exon 5, TGFBR2	1 month	/
Kuppler KM et al	1583G > A (R528H), exon 7, TGFBR2	3 months	/
Wisniewski K et al	/	1 month	/
Valenzuela I et al	c.1381 T > C, TGFBR2	1 month	Arthrogryposis
Ozawa H et al.; Kawazu Y et al	598A > C, TGFBR1	Prenatal ultrasound (36 weeks)	/
Viassolo V et al	TGFBR2	Prenatal ultrasound (19 weeks)	/
Yetman AT et al. [1]	1583G > A, exon 7, TGFBR2	1 month	Beals syndrome, Larsen syndrome, arthrogryposis
Yetman AT et al. [2]	1570G > A, exon 7, TGFBR2	1 month	Larsen syndrome
Yetman AT et al. [3]	1318G > A, exon 5, TGFBR2	9 years	Larsen syndrome
Yetman AT et al. [4]	865-873delACAGAGAAG, exon 4, TGFBR2	1 month	Arthrogryposis, Larsen syndrome
Yetman AT et al. [5]	IVS5-1G > A (splicing variant), TGFBR2	6 months	Beals syndrome
Chung BHY et al	1583G > A(R528H), exon 7, TGFBR2	1 month	/
Patient 1	c.1378C > G, p (Arg460Gly), exon 5, TGFBR2	1 month	Loeys Dietz
Patient 2	c899G > A, p (Arg300Gin), TGFB3	/	/

Although the exact timing of the diagnosis of LDS was not specified in most of the papers, it can be inferred that most of them were made in the very first years or even months of life, due to the precious appearance of systemic symptoms and clinical appearance. For instance, in 15 patients (93.7%) vascular abnormalities have been identified in the first year of life. In 3 subjects (18.7%) the initial recognition was through prenatal ultrasound, and in 2 individuals LDS was identified only post-mortem.

Genetic analyses are precisely described in 14 out 16 patients. *TGFRB2* mutations were reported in 12 individuals (75%), while *TGFBR1* and *TGFB3* were involved in just one subject each. Four patients presented a guanine to adenine substitution (1583G > A) on exon 7 of *TGFBR2* gene.

As for the differential diagnosis, arthrogryposis and Larsen syndrome were hypothesized as the original diagnosis in 4 cases each, while Beals syndrome in 2 cases.

14/16 individuals (87.5%) were alive at the time of the report. Of the 2 patients with negative outcome, one died at 21 days of life due to massive bleeding from the thoracic cavity caused by rupture of the right pulmonary artery, despite undergoing a bilateral artery bending at 9 days of age. The second one deceased at 9 years of age due to aortic dissection.

In terms of neonatal anthropometric measures, 4 subjects (25%) were small for gestation age (SGA) and 2 (12.5%) were large for gestational age (LGA).

Table 3 describes the clinical phenotype of the patients, including vascular and cardiac abnormalities.

**Table 3:** Phenotypic description of the patients. “Cardio” indicates any structural abnormality of the heart, such as septal defects or cardiomegaly. “Neuro” includes any neurological abnormality on physical examination, such as hypotonia. “Skeletal” includes any bone or joints alterations (including arachnodactyly) apart from spine involvement, which is reported as “spine”. Head includes dysmorphic features of the patient’s face, with the exclusion of micrognathia (“micrognathia”) and cleft palate (“uvula/palate”)

Author	Aorta	Pulmonary	Tortuosity	Cardio	Skeletal	Eyes	Head	Uvula/Palate	Micrognathia	Spine	Hernias	Feet	Neuro
Choo JTL et al	Yes	Yes	/	Yes	Yes	Yes	Yes	Yes	Yes	/	/	/	Yes
Ilyn VN et al	Yes	/	Yes	Yes	/	Yes	/	Yes	/	/	/	/	/
Muramatsu Y et al	Yes	Yes	Yes	Yes	Yes	Yes	/	Yes	Yes	/	Yes	Yes	Yes
Kuppler KM et al	Yes	Yes	/	/	Yes	Yes	/	Yes	/	/	/	Yes	/
Wisniewski K et al	Yes	Yes	Yes	Yes	Yes	Yes	/	Yes	Yes	/	Yes	/	/
Valenzuela I et al	Yes	/	Yes	/	Yes	Yes	/	Yes	/	/	Yes	Yes	/
Ozawa H et al	Yes	Yes	Yes	Yes	Yes	/	/	Yes	/	/	/	/	/
Viassolo V et al	Yes	/	/	/	Yes	Yes	Yes	Yes	Yes	/	/	Yes	/
Yetman AT et al. [1]	Yes	Yes	Yes	Yes	Yes	Yes	Yes	Yes	/	Yes	Yes	Yes	Yes
Yetman AT et al. [2]	Yes	Yes	Yes	Yes	Yes	Yes	Yes	Yes	/	Yes	Yes	Yes	Yes
Yetman AT et al. [3]	Yes	/	Yes	/	Yes	Yes	Yes	Yes	/	Yes	Yes	Yes	Yes
Yetman AT et al. [4]	Yes	Yes	Yes	Yes	Yes	Yes		Yes	/	Yes	Yes	Yes	Yes
Yetman AT et al. [5]	Yes	/	/	/	Yes	Yes	Yes	Yes	/	Yes	Yes	Yes	Yes
Chung BHY et al	Yes	Yes	Yes	/	Yes	/	/	/	Yes	Yes	Yes	Yes	/
Patient 1	Yes	/	/	Yes	Yes	Yes	Yes	/	Yes	/	/	/	Yes
Patient 2	/	/	/	/	Yes	Yes	Yes	Yes	Yes	/	/	/	/

The aorta was involved all but one patient (93.7%), mainly in its ascending component (75%) with a significant dilation or aneurysm, while the aortic arch was involved in 2 cases (12.5%). One patient presented an aortic coarctation and another one an interrupted aortic arch. The pulmonary circle was also frequently involved, since 9/16 patients (56.2%) presented some degree of dilation in one of its main arteries. Other vessels affected were the ductus arteriosus, in which an aneurysm was found in 2 patients (12.5%), the subclavian artery and carotid and vertebral artery, one time each. Arterial tortuosity was reported in 10/16 subjects (62.5%).

As for the cardiac involvement, patent ductus arteriosus was identified in 7 patients (43.7%), atrial septal defect and ventricular sept defect in 2 patients each (12.5%), cardiomegaly and congestive heart failure in 3 (18.7%), double outlet right ventricle in one. Overall, a cardiac murmur was reported in just 3 patients (18.7%).

All the patients except one (93.7%) presented one or more abnormalities of the bone or the joints. Arachnodactyly was described in 7 subjects (43.7%), articular contractions (such as camptodactyly and clenched hands) in 10 (62.5%), joint laxity with or without dislocations in 10 (62.5%), pectoral deformity in 2 (12.5%), marphanoid appearance, syndactyly, and absent distal phalanges in one patient each. Club feet were reported in 10 patients (62.5%), metatarsus abductus and genu recurvatum in 2 (12.5%). Translucent and thin skin was reported in 4 subjects (25%).

As for the head and eyes’ involvement, 13 patients (81.2%) presented with hypertelorism, 6 (37.5%) had strabismus and blue sclerae, 5 (31.2%) had macrocephaly and 2 (12.5%) had frontal bossing. Only one patient had a craniosynostosis. Occasionally, other clinical features were also described, such as palpebral downslanting, low set ears and deep-set eyes.

Bifid or broad uvula and/or cleft palate were present in 14 subjects (87.5%), while micro and/or retrognathia characterized 7 of them (43.7%).

Cervical spine abnormalities, especially vertebral subluxations, were described in 6 patients (37.5%). Congenital scoliosis was described in one patient. Hernias, whether inguinal or umbilical, appeared in 9 patients (56.2%).

Lastly, hypotonia, diffuse or marked, was reported in 6 patients (37.5%), and neurodevelopmental delay in 3 (18.7%).

In terms of treatment, 10 patients (62.5%) started a medical therapy (Table 4). Overall, angiotensin II receptor blockers (ARBs) were the most frequently used (31.2%), followed by ACE-inhibitors and beta-blockers (25% each). 3 patients required more than one drug. 3 patients remained clinically stable under medical treatment, and one even improved while taking a losartan.

**Table 4:** Therapeutic management. ARB: angiotensin II receptor blocker (losartan). PDA: patent ductus arteriosus. ASD: atrial sept defect. VSD: ventricular sept defect. NR: not reported

Report	Medical therapy	Surgery	Surgical treatment	Surgical timing (age)
Choo JTL et al	/	No	/	/
Ilyn VN et al	Beta-blocker	Yes	Aneurism resection, repair of aortic coarctation, subclavian ligation	1 year
Muramatsu Y et al	ACE-inhibitor	Yes	Pulmonary artery banding - ASD and VSD closure, pulmonary artery plastic	12 days - 42 days
Kuppler KM et al	/	No	/	/
Wisniewski K et al	Beta-blocker, ARB	Yes	PDA closure - Valve sparing aortic root replacement	40 days - 8 months
Valenzuela I et al	Beta-blocker, ARB	No	/	/
Ozawa H et al	ARB	Yes	Bilateral pulmonary artery banding	9 days (urgent)
Viassolo V et al	/	No	/	/
Yetman AT et al. [1]	Beta-blocker, ACE-inhibitor	No	/	/
Yetman AT et al. [2]	ACE-inhibitor	Yes	PDA ligation	NR
Yetman AT et al. [3]	/	Yes	Aortic valve replacement, aortic replacement	9 years
Yetman AT et al. [4]	ACE-inhibitor	Yes	Valve sparing surgery	2 years
Yetman AT et al. [5]	/	Yes	Valve sparing surgery	3 years
Chung BHY et al	ARB	No	/	/
Patient 1	ARB	No	/	/
Patient 2	/	No	/	/

Eight patients (50%) underwent surgical procedure. The type of surgical management and the surgical timing are all reported in Table 4.

## Conclusions

LDS is a rare autosomal dominant condition, and its neonatal presentation is even rarer. However, its precocious cardiovascular phenotype, sometimes recognizable even with prenatal ultrasound, might be extremely severe and put a newborn’s life in danger since its very first days of life. Hence, LDS is a disorder that pediatricians and neonatologists should be able to suspect. We suggest that in a newborn or a fetus with abnormalities compatible with connective tissue disorders, and even more if vascular abnormalities are precociously detected, Loeys-Dietz syndrome should be the first diagnosis to take into consideration. Moreover, as our review of the literature suggested, when arthrogryposis is suspected early in life or even prenatally, it is reasonable to include LDS in the differential diagnosis process. Benefits of an early and specific diagnosis of LDS are various and imply a potential modification of the natural history of the disease with early interventions on its complications.

To our knowledge, the first case described in our paper is the only clinical report of an LDS clinically suspected and a molecularly confirmed within the first month of life. Of course, the in-trio- WES approach has significantly speeded up the diagnostic process and its cost-effectiveness in the neonatal clinical setting is undeniable. In that case was detected a novel variant in *TGFBR2*. This gene encodes for a key TGF-beta transmembrane receptor, regulating cell proliferation and differentiation and extracellular matrix production. Most of the already described pathogenic *TGFBR2* LDS variants are located in the intracellular kinase domain where is also located the herein reported variant. Interestingly, another variant involving the same nucleotide and responsible of a different aminoacidic modification (p.R460C) has been reported as responsible of isolated familial thoracic aortic aneurysms and dissections, Marfan-syndrome related disorder and rarely to classic LDS [32–34]. Moreover, other two variants in the following nucleotide, but involving the same amino acid (p.R460H, p.R460L) have been described, highlighting that the amino acid 460 is strategically located within a highly conserved region of the protein domain and its substitutions interfere with the receptor’s ability to transduce signals [17–28]. For this reason, the detected variant has been considered as pathogenic (according to the *ACMG* guidelines).

The importance of an early diagnosis of LDS relies on the possibility of tackling the otherwise inevitable progression of its vascular abnormalities with a medical therapy. As discussed above, various drugs have shown some degree of efficacy in preventing the evolution of aneurysms to rupture or dissection in connective tissue disorders. Although there’s still small evidence of this efficacy in LDS, essentially because of the lack of dedicated clinical trials, the possible benefits of beta-blockers or ARBs should encourage their immediate start when vascular abnormalities are detected. This is even more true considering that both drugs have a positive safety profile in newborns and young children, with very few adverse effects. Delaying a pharmacological approach could allow a progression of the vascular lesions and lead to cardiac symptoms, congestive heart failure, and to the need of early surgery. Although, as reported previously in Table 4, most of the surgical procedures described in the literature occurred without complications, LDS subjects present a peculiar vascular fragility that put them at higher risk of vessel rupture. Ideally, surgery should be performed only on those subjects who did not respond to a medical therapy, or on subjects with high-risk vascular abnormalities. Moreover, considering that aortic tissues from affected patients show evidence of increased TGFβ signaling, new personalized options for the implementation insisting on this pathway are worth to consider [35].

As described in the second case report, LDS features might not be evident from the very first days of life, since the syndrome’s phenotype is extremely broad. On that occasion, the key to the precocious diagnosis was the family history highly suspicious for connective tissue disorders. Based on these premises, we suspect that LDS might be underdiagnosed especially during childhood, when marphanoid features can be less clear. A suggestive family history, even if in presence of mild dysmorphisms, can be crucial to start an appropriate genetic evaluation. In fact, about one third of LDS cases are familiar and generally characterized by milder clinical signs than sporadic cases. Indeed, the variant identified in the second case has already been reported in patients with isolated thoracic aortic disorders and a mild spectrum of LDS [36, 37].

Finally, we hope that new clinical trials will be conducted to finally assess the utility of the already used medical therapy in LDS patients, and to identify new molecules and targets that may be more efficient in preventing the progression of vascular abnormalities.

## Data Availability

All data generated or analyzed during this study are included in this published article and its supplementary information files.

## Abbreviations

LDS: Loeys Dietz syndrome
TGFB: Transforming growth factor beta
TGFBR: Transforming growth factor beta receptor
SMAD: Small mother against decapentaplegic
ACE: Angiotensin converting enzyme
ARB: Angiotensin II receptor blockers
MS: Marfan syndrome
WES: Whole exome sequencing
SGA: Small for gestational age
LGA: Large for gestational age
ACMG: American College of Medical Genetic and Genomics
